# A Novel Renal Manifestation in *GLIS3*-Related Disorder in a Pediatric Patient With Cystic Nephroma

**DOI:** 10.14740/jmc5300

**Published:** 2026-06-03

**Authors:** Ibrahim Alharbi, Danah Khalid Alharbi, Razan Abdelrahim, Bayan Fawaz Alzahrani

**Affiliations:** a Department of Pediatrics, Faculty of Medicine, Umm AlQura University, Mecca, Saudi Arabia; b Faculty of Medicine, Umm Al-Qura University, Makkah, Saudi Arabia

**Keywords:** *GLIS3* mutation, Neonatal diabetes mellitus, Congenital hypothyroidism, Renal mass, Benign renal tumor

## Abstract

A 10-year-old Saudi girl with a homozygous *GLIS3* gene deletion presented with the classic triad of neonatal diabetes mellitus, congenital hypothyroidism, and congenital glaucoma. During routine surveillance, an incidental complex left renal mass was identified. Comprehensive imaging and histopathological analysis following a core needle biopsy diagnosed a benign multicystic nephroma with features of a mixed epithelial and stromal tumor (MEST). This represents a novel renal manifestation, expanding the phenotypic spectrum of *GLIS3*-related disorders beyond simple cystic dysplasia. Management favored active surveillance over surgical resection given the lesion’s benign nature. The case underscores the necessity for proactive, multidisciplinary, and lifelong monitoring in patients with *GLIS3* mutations, who are at risk for evolving multisystem complications, including atypical renal neoplasms, into adolescence and adulthood.

## Introduction

GLIS3 belongs to the GLIS subfamily of Kruppel-like zinc finger proteins and functions as a transcription factor that activates or represses gene transcription [1, 2]. The *GLIS3* gene is located on chromosome 9p24.2 and regulates gene expression during early embryonic development, ensuring cell differentiation and organ formation [1, 2]. GLIS3 plays an important role in the development and maturation of the pancreas, thyroid, liver, kidneys, and eyes [3, 4]. Mutations in *GLIS3* can alter gene expression in these organs and cause congenital disorders, including neonatal diabetes mellitus (NDM), congenital hypothyroidism (CH), polycystic kidney disease, and liver fibrosis. The impact of these mutations depends on how they disrupt gene activation or repression [1, 3, 4]. Studying these mutations reveals the underlying mechanisms of these multisystem diseases and offers insight into potential therapeutic targets [5].

*GLIS3*-related neonatal diabetes is very rare. Fewer than 100 cases are reported worldwide. Middle Eastern and South Asian populations appear to have a higher frequency, possibly due to higher rates of consanguineous marriages [6–8]. Clinicians rely on genetic testing to diagnose cases. This is especially important for newborns with insulin-deficient hyperglycemia, CH, kidney cysts, or liver problems. To confirm a diagnosis, geneticists identify biallelic pathogenic *GLIS3* variants, either homozygous or compound heterozygous [1, 2]. Once established, providers recommend evaluations such as thyroid function tests, renal ultrasound, and liver tests to assess multisystem involvement [6, 9].

No cure currently exists for *GLIS3*-related neonatal diabetes, so clinicians focus treatment on managing symptoms. They initiate early insulin therapy for hyperglycemia, prescribe levothyroxine for hypothyroidism, and monitor or target therapies for liver or kidney involvement [3, 6]. A multidisciplinary team, including genetic counselors, supports high-risk populations because the severity of organ involvement largely determines outcomes [6, 9].

This case underscores the need for shared international expertise to optimize the management of *GLIS3* mutations.

## Case Report

We present our case, a 10-year-old Saudi girl with a *GLIS3* deletion (9p24). She was diagnosed in October 2020. Diagnosis was based on genetic testing (microarray). She presented for regular follow-up and for imaging. She had an incidental finding of a renal mass on abdominal ultrasound. She was admitted to the pediatric ward as a case of *GLIS3* deletion with a left renal mass for further investigations. After taking a focused history, there was no flank pain or hematuria. There was no fever, loss of appetite, weight loss, fatigue, or night sweats. There was no vomiting, diarrhea, or constipation, no loss of consciousness.

Past medical history is significant for multiple conditions. She has congenital type 1 diabetes mellitus and is on insulin therapy, including insulin degludec (22 units once daily) and insulin aspart (5–6–6 units per day, subcutaneously). In addition, she has CH, and is on thyroxine (125 µg once daily for 5 days and 150 µg once daily for 2 days). She also suffers from congenital glaucoma (CG) and cone-rod dystrophy, for which she uses bimatoprost 0.03% and timolol 0.5%, one drop at night daily.

On perinatal history, she was a late-premature newborn delivered at a gestational age of 36 weeks. She was delivered via vaginal delivery as a late-premature due to intrauterine growth restriction (IUGR). Vaccinations are up to date. She is developmentally following her age. Family history showed that her older sister was diagnosed with the same condition. There was positive consanguinity. Premarital testing was compatible.

Upon examination, she looked well, was vitally stable, and was afebrile, with an oxygen saturation of a 100% on room air. Growth parameters showed a weight of 35.2 kg and a height of 148 cm, both at the 50th percentile and appropriate for her age. The patient was conscious and alert. Her pupils were equal and bilaterally reactive. No lymphadenopathy was noted. Chest examination revealed equal air entry bilaterally, with no added sounds. The abdomen was soft and non-tender, with no palpable masses.

Investigations showed that the complete blood count (CBC) was normal. Inflammatory markers showed a C-reactive protein of 4 mg/L. Renal function tests (RFTs) showed the following: urea level of 4.3 mg/dL (within the normal range), creatinine 72 µmol/L (elevated, normal range: 27–54 µmol/L). Creatinine was initially elevated, indicating renal injury, but it normalized after admission with appropriate hydration. CO_2_ was 24 mmol/L, anion gap 9 mmol/L, Na 137 mmol/L, K 4.3 mmol/L, and Cl 104 mmol/L. Liver function test (LFT) showed that aspartate transaminase (AST) was 23 U/L, alkaline phosphatase (ALP) 333 U/L, and lactate dehydrogenase (LDH) 250 U/L. Coagulation profile revealed partial thromboplastin time (PTT) of 40.7 s, prothrombin time (PT) of13.6 s, and international normalized ratio (INR) of 0.99. Blood culture was negative.

Imaging included a renal ultrasound, which showed that the left kidney measured 9.2 cm with mild hydronephrosis and a dilated renal pelvis measuring 4.9 cm. A renal mass was identified, measuring 4.1 × 3.7 cm, with multiple echogenic foci noted at the periphery, measuring 0.4 cm and 0.3 cm. It showed a well-defined, heterogeneously echogenic lesion with central hypoechogenicity in the interpolar region, with no internal vascularity.

**Figure 1:**
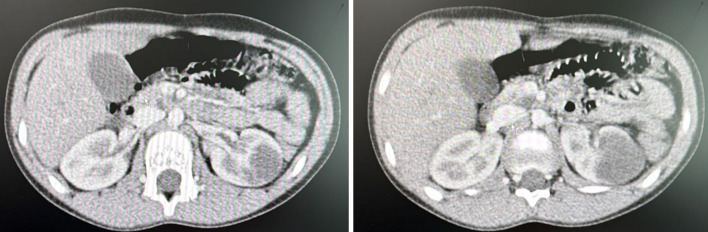
CT showing a left kidney mass (arrow). CT: computed tomography.

**Figure 2:**
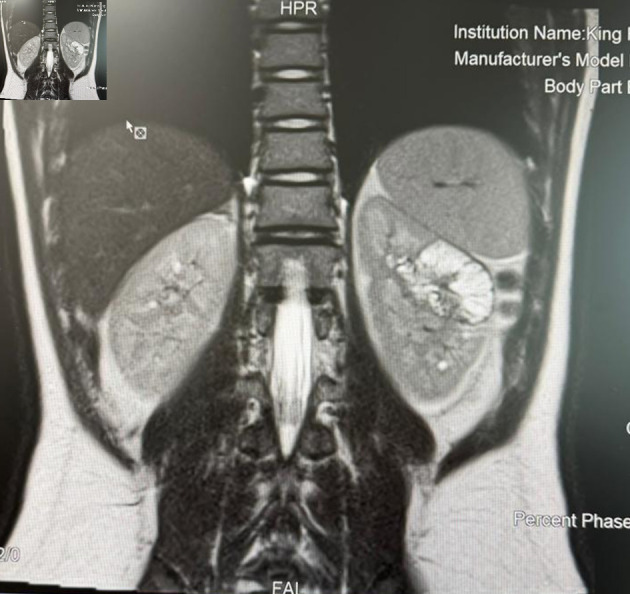
MRI showing a left kidney mass (arrow). MRI: magnetic resonance imaging.

Abdominal computed tomography (CT) with contrast showed a hypodense left renal mass (Fig. 1). Assessment of the pancreatic head showed two cystic structures. Magnetic resonance imaging (MRI) with intravenous (IV) contrast showed a left renal mid-polar mass (Fig. 2) measuring about 3.9 × 3.6 × 3.6 cm. The lesion demonstrated multiple septations and signal voids denoting tortuous vessels. It was hyperintense on T2 images and hypointense on T1 images, with focal areas of increased intensity likely representing blood degradation products. There were faint restricted diffusion and focal areas of cystic change, exhibiting gradual postcontrast enhancement mainly involving the septations. In addition, a multiloculated complex cystic lesion was seen exophytic at the pancreatic head/uncinate process and measuring about 1.7 × 1.1 × 1.8 cm (transverse, anteroposterior, and craniocaudal dimensions). There was evidence of a small nodule measuring approximately 3 mm that demonstrated no obvious postcontrast enhancement. Another smaller cystic lesion was noted in the pancreatic neck, measuring 0.9 × 0.4 cm.

Bone scan showed a multiple focal tracer uptake seen at the right iliac crest, as well as the spinous process of the L3 vertebra. Upon these significant findings, she was admitted as a case of a left renal mass for further investigation. The differential diagnoses included lymphoma and, less likely, other conditions such as Wilms tumor and/or renal cell carcinoma.

A multidisciplinary team was consulted (pediatric hematology–oncology, nephrology, anesthesia, and interventional radiology). A left renal mass biopsy was recommended and performed, and the specimen was subsequently submitted for histopathologic evaluation. The morphologic and immunohistochemical profile of the core biopsy is suggestive of a multicystic nephroma (MCN) and a mixed epithelial and stromal tumor (MEST). Pediatric cystic nephroma is a distinct entity that is associated with *DICER1* mutations.

Microscopic findings were as follows. The section showed a core of lesional tissue composed of variably sized cysts lined by bland cuboidal epithelium. The fibrous stroma showed variable spindle cells, few differentiated tubules, and chronic inflammatory cells. No atypia was seen, and no blastemal elements were identified in the tissue examined. Immunohistochemistry was performed, and all controls showed appropriate reactivity. The epithelial cells are positive for paired box gene 8 (PAX8) and GATA-binding protein 3 (GATA3). The stromal cells are positive for estrogen receptor (ER) and smooth muscle actin (SMA). Inhibin, desmin, racemase, and carbonic anhydrase IX are negative.

All findings, in correlation with her clinical picture—including the absence of constitutional symptoms and the histopathology report—suggest a benign left renal mass. The consensus favored observation and frequent follow-up over immediate interventions, such as surgical resection.

## Discussion

This case presents a 10-year-old Saudi girl with a confirmed *GLIS3* deletion, adding a significant new dimension to the evolving clinical narrative of *GLIS3*-related disorders. Her presentation aligns with the classic triad of NDM, CH, and CG. The incidental discovery of a complex renal mass expands the phenotypic spectrum and reveals critical management dilemmas.

First described as a syndromic cause of NDM and CH, *GLIS3*-related disorders are now recognized as a complex multisystem condition with variable expressivity [1, 10]. The cardinal features present in our patient—persistent insulin-dependent diabetes diagnosed in the neonatal period, primary hypothyroidism requiring levothyroxine, and CG—are consistent with those reported in over 22 cases to date [11, 12]. Our patient’s positive family history and parental consanguinity are also recurrent themes, reflecting the autosomal recessive inheritance pattern and the higher prevalence reported in Middle Eastern populations due to founder effects and consanguineous marriages [10, 13]. Beyond this classic triad, the phenotypic spectrum is remarkably broad. As established in the large cohort by Dimitri et al (2015), systemic involvement can include hepatic fibrosis, cholestasis, polycystic kidney disease, exocrine pancreatic insufficiency, developmental delay, sensorineural deafness, osteopenia, and skeletal anomalies [14]. Interestingly, while hepatic and renal parenchymal diseases are common, their severity and progression are highly variable, even among patients with identical mutations [14, 15]. Our patient showed this variability: she had no clinical or biochemical evidence of significant liver disease at presentation, but her renal ultrasound revealed significant pathology. This underscores the principle that the absence of one classic feature (e.g., severe liver disease) does not preclude the development of another (e.g., complex renal disease).

The collected literature on *GLIS3* mutations indicates that disease severity is associated strongly with both the number of exons deleted and the nature and site of the deletion. Large deletions involving multiple exons—particularly those spanning central coding regions (e.g., exons encoding the zinc-finger DNA-binding domains)—are typically correlated with more severe, multisystem phenotypes, including neonatal diabetes, CH, hepatic fibrosis, and renal cystic disease. Reported deletions often cluster within critical genomic intervals on chromosome 9p24.2, and although exact genomic coordinates vary between cases, pathogenic variants frequently disrupt exons encoding the GLIS3 protein’s functional domains. When deletions eliminate multiple contiguous exons or cause frameshifts, they are expected to produce shortened or unstable proteins with extensive damage of transcription factor activity. In contrast, smaller deletions or mutations affecting lesser exons may allow partial protein function, leading to milder or more variable phenotypes. Functionally, specific exons within *GLIS3* are vital for encoding the Kruppel-like zinc finger motifs responsible for DNA binding and transcriptional regulation in pancreatic β-cells and thyroid tissue; therefore, their loss directly impairs insulin gene expression and endocrine development.

In general, the genotype–phenotype relationship suggests that both the extent of exon loss and the functional importance of the affected exons determine the clinical severity through their effect on protein integrity and transcriptional activity.

Renal involvement in *GLIS3* mutations typically manifests as cystic dysplasia, enlarged kidneys, or loss of corticomedullary differentiation [14, 16]. However, the discovery of a well-defined, complex, and renal mass in our patient represents a new and clinically significant finding. The histopathological diagnosis of an MCN with features overlapping MEST moves beyond simple renal cysts and introduces a neoplastic element to the GLIS3 phenotype. The pathogenesis of this lesion may be attributed to the fundamental role of GLIS3 in renal development. GLIS3 is highly expressed in the kidneys and interacts with pathways critical for nephron differentiation and tubulogenesis [17]. In animal models, *Glis3* deficiency leads to polycystic kidney disease, suggesting its role in maintaining tubular epithelial integrity and regulating cystic disease pathways [17, 18]. It is reasonable that the loss of GLIS3 function creates a permissive cellular milieu prone to dysplastic and hyperplastic growth, potentially culminating in benign neoplastic formations like the one observed. Furthermore, the identification of pancreatic head cysts on MRI in our patient is consistent with the findings of Dimitri et al, who reported pancreatic cysts in siblings with a *GLIS3* deletion, reinforcing the gene’s role in pancreatic ductal architecture [14].

Importantly, pediatric cystic nephroma is a different issue strongly associated with DICER1 syndrome, a tumor predisposition disorder [19]. While our biopsy suggested MCN, the immunohistochemistry profile and the patient’s known *GLIS3* deletion make *DICER1*-related neoplasm less likely. This case, therefore, introduces a critical diagnostic consideration. In patients with *GLIS3*-related disorders, newly detected renal masses should not be automatically presumed malignant (e.g., Wilms tumor) but should undergo thorough histopathological evaluation, as a benign *GLIS3*-related dysplastic or neoplastic process may be present.

The management of our patients’ renal mass illustrates a standard shift towards conservative, surveillance-based strategies for certain *GLIS3*-related complications. Given the benign histology, absence of constitutional symptoms, and the significant morbidity of nephron-sparing or radical nephrectomy in a child with a potential for progressive bilateral renal disease, the multidisciplinary consensus for active observation was prudent. This approach aligns with the management of other benign cystic renal lesions in childhood and prioritizes preservation of renal function [20]. This case underscores the necessity of a proactive, multidisciplinary follow-up protocol for GLIS3 patients. Our recommended surveillance model includes an annual renal ultrasound, with close monitoring of the existing mass and the contralateral kidney. This should be accompanied by periodic assessment of renal function (creatinine, cystatin C) and blood pressure. Also, biannual liver function tests and periodic abdominal ultrasound are recommended to monitor for delayed-onset fibrosis or portal hypertension, as seen in other cases [14, 21]. Monitoring for signs of exocrine pancreatic insufficiency (steatorrhea, poor weight gain) should be performed, with consideration of fecal elastase testing. Surveillance of pancreatic cysts is advised. Continued strict management of diabetes (glycated hemoglobin (HbA1c) targets) and hypothyroidism is essential, with awareness of possible thyroid-stimulating hormone (TSH) resistance requiring higher levothyroxine doses [11, 22], along regular audiological, ophthalmological, and developmental assessments.

The prognosis in *GLIS3*-related disorders is essentially linked to the extent of multisystem involvement, mostly liver and kidney disease [11, 14]. Our patient, now in her second decade, represents a more favorable trajectory compared to cases with infantile mortality from hepatic failure or sepsis [1, 14]. The specific mutation may influence outcomes. For example, Senee et al (2006) assumed that tissue-specific transcripts might explain phenotypic variability, where larger deletions sparing certain exons could result in milder organ involvement [1]. Our patient’s microarray-confirmed deletion of 9p24.2 (encompassing exons 10–11) is identical to the mutation reported in a Yemeni female who developed hepatic fibrosis and exocrine insufficiency [14].

Additionally, our patient has not (yet) manifested these features, highlighting the imperfect genotype–phenotype correlation and the potential influence of genetic modifiers or environmental factors. The oldest surviving patients reported, including a 36-year-old adult and the 17-year-old Turkish male from Sarikaya et al (2023), similarly lacked severe parenchymal organ disease, indicating that the absence of progressive hepatic or renal failure may be a key determinant for survival into adulthood [11]. Our patient’s current clinical stability offers cautious optimism but mandates vigilance for the late-onset complications documented in the literature.

### Conclusions

This case of a Saudi child with a *GLIS3* deletion reinforces the classic endocrine features of the syndrome while dramatically expanding its renal phenotypic spectrum to include complex, benign cystic neoplasms. The incidental finding of an MCN underscores that *GLIS3*-related disorders are not static diagnoses but evolving diseases requiring lifelong, multidisciplinary surveillance. Management must be individualized, balancing intervention for acute complications with a conservative, monitoring-based approach for non-life-threatening manifestations to preserve organ function. This report adds to the growing evidence that patients with *GLIS3* mutations can survive into later childhood and beyond, and it calls for the development of standardized international surveillance guidelines to optimize their long-term outcomes.

## Data Availability

The authors declare that data supporting the findings of this study are available within the article.
